# Reflections on the COVID-19 Pandemic

**DOI:** 10.1590/S1677-5538.IBJU.2020.04.02

**Published:** 2020-05-20

**Authors:** Francisco J. B. Sampaio

**Affiliations:** 1 Int Braz J Urol Rio de JaneiroRJ Brazil Emeritus Editor, Int Braz J Urol , Rio de Janeiro , RJ , Brazil ;; 2 National Academy of Medicine, Rio de JaneiroRJ Brazil Past President, National Academy of Medicine, Rio de Janeiro , RJ , Brazil

This editorial represents a compilation of reflections that I have made during the last few days. Now, at the invitation of the Chief Editor I would like to share them with the urological community.

After the Covid-19 pandemic recedes, humanity will need to rethink their way of living. Everyone is vulnerable. The virus doesn’t distinguish: the rich or the poor, the important or the ordinary people. Everyone now realizes there is no point in having money or residences in three or four different countries or on various continents in an attempt to escape the crises; they can’t escape, the world becomes too small. Everyone is at risk: politicians, ministers, governors, prime ministers, presidents, princes, the rich and the poor ... no one can escape the virus, and at this moment, just being able to breathe well is a blessing.

Urologists are no longer operating; elective surgeries have been postponed, outpatient clinics have suspended consultations, and in many countries urologists have already left the specialty and are dedicating themselves to patients with Covid-19. The healthcare systems of many countries are collapsing and the percentage of doctors and other health professionals who have become contaminated is very large.

I hope that when this pandemic passes, all countries will start to worry about the many other infectious diseases, which have been plaguing mankind for years, even though treatments and/or vaccines exist for many of them. Why has the world never been so concerned with yellow fever, rabies, measles, meningitis, cholera, malaria, whooping cough, rotavirus, shigellosis, hepatitis B and tuberculosis, as well as many other neglected contagious infectious diseases that kill so many? As example, tuberculosis alone kills 4,500 people a day in the world. Nevertheless, these diseases are not pandemics.

We are in panic because Covid-19 is very contagious and does not discriminate. Everyone can get sick and very quickly fill the hospitals, and then, no one, not even the rich and powerful can access the lifesaving care they desperately need. Covid-19 also causes an enormous economic chaos, affecting all social classes, no one is immune. Since Covid-19 does not distinguish, and has no treatment, the World is now in a panic.

At this moment we still don’t know how to deal with this disease. Covid-19 has no vaccine and no effective proven treatment. It seems that the only effective way to reduce the devastating effects of Covid19 on healthcare systems is social distancing and isolation. The problem is social distancing also leads to economic collapse and it is difficult to recover from the effects of this form of treatment.

In the meantime, scientists around the world are desperately searching for effective vaccines and treatments for this devastating disease and we are all hoping that they will soon emerge to mitigate this pandemic. The world needs to work together to both defeat is as well as finding a solution.

At this point, we must emphasize the following advice to the population: **stay at home** . The fewer people who became ill in the short term, the better the health systems will be able to care for patients with Covid-19 in serious condition, thus increasing the chance of survival. Be well.

Respectfully,

